# Tobacco use and knowledge of lung disease risks in medical students: Implications for medical education

**DOI:** 10.12669/pjms.42.3.13611

**Published:** 2026-03

**Authors:** Zeliha Demir Giden

**Affiliations:** 1 Zeliha Demir Giden Department of Chest Diseases, Faculty of Medicine, Harran University, Sanliurfa, Turkey

**Keywords:** Smoking, Risk factors, Lung disease, Medical student

## Abstract

**Objective:**

Smoking is one of the most important causes of preventable deaths and plays a major role in the development of various pulmonary diseases. This study aimed to evaluate the smoking habits and knowledge levels of medical students in clinical training regarding smoking-related lung diseases.

**Methodology:**

This study was designed as a cross-sectional, questionnaire-based survey. A total of 290 volunteer medical students in their fourth, fifth, and sixth years of education at Harran University Faculty of Medicine were included in the study. Participants were administered a questionnaire inquiring about sociodemographic characteristics, smoking status, and smoking-related lung diseases. Data were analyzed using the SPSS program, and the chi-square test was applied for categorical variables.

**Results:**

The mean age of the students was 23.57±1.82, and 30.3% were smokers. The smoking rate was higher among males (75% male, 25% female) and increased with grade level (highest in 6th grade). Most students recognized the relationship between smoking and lung cancer (93.8%) and COPD (93.4%), while fewer associated smoking with asthma (70.0%), interstitial lung diseases (63.4%), pneumonia (61.4%), pneumothorax (52.8%), and tuberculosis (42.1%). A significant difference among the class levels was observed only in the recognition of the smoking–pneumothorax relationship (p<0.05).

**Conclusions:**

Cigarette smoking is high among medical students and increases particularly in the upper classes. Although the relationship between smoking and lung cancer and COPD is well known, there are gaps in knowledge regarding its relationship with other lung diseases. More comprehensive and structured education on the health consequences of smoking should be integrated into medical training to better prepare future physicians for tobacco control.

## INTRODUCTION

Tobacco is a plant in the nightshade family, also known as Solanaceae, that is generally annual, though in some species it is perennial. Tobacco is distinguished from other plants by the presence of nicotine, an organic nitrogen-containing substance, in its leaves. Nicotine is a potent alkaloid that is addictive and euphoric. Cigarettes are tobacco products prepared by rolling shredded tobacco in thin paper, usually cylindrical in shape, with or without a filter on one end.^1^,^2^ Approximately 5.4 million people die each year due to smoking, and smoking is ranked first among preventable deaths by the World Health Organization (WHO). If necessary measures are not taken, it is estimated that the number of deaths caused by smoking will reach eight million annually worldwide before 2030. Smoking has many negative effects on human health, including its toxicity, inflammatory effects, mutagenic effects, and carcinogenic effects.^3^ Tobacco-related lung diseases encompass a wide range of conditions, from the very common lung cancer and chronic obstructive pulmonary disease (COPD) to the rare interstitial lung disease group, including Langerhans cell histiocytosis (LCH).^2^

It is important for physicians to have knowledge about tobacco-related diseases. In addition, their recognized status in society as health experts, their impartiality, and their ability to provide advice to patients impose significant responsibilities on physicians in tobacco control. Evaluating the knowledge level of clinical internship students is important in terms of understanding their awareness and role in preventing smoking-related lung diseases in their future medical practice. The rate of smoking among physicians can be considered an indicator of the effectiveness of the tobacco control program in that country. Conversely, in a country where physicians smoke at high rates, it will be difficult to convince the public about the harmful effects of smoking. The prevalence of smoking among physicians has markedly declined in many developed Western countries.^2^ Studies from Turkey and other regions have shown that smoking prevalence tends to increase during medical education, particularly in the later academic years, possibly due to academic stress, increased workload, and social factors. However, most of these studies have primarily focused on smoking prevalence and attitudes, rather than detailed knowledge of smoking-related lung diseases.^4^,^5^ Therefore, synthesizing findings from previous studies highlights the need for a more comprehensive evaluation of both smoking behavior and disease-specific knowledge among medical students. The present study aimed to assess smoking prevalence and level of knowledge of smoking-related lung diseases among clinical-year medical students. Thereby contributing to the existing literature and providing evidence to support the integration of structured and longitudinal tobacco-related education into undergraduate medical.

## METHODOLOGY

This study was designed as a cross-sectional, questionnaire-based survey. Voluntary participants were recruited from fourth, fifth, and sixth year medical students enrolled at Harran University Faculty of Medicine. A total of 290 medical students participated in the study, including 120 (41.4%) fourth year, 80 (27.6%) fifth-year, and 90 (31.0%) sixth-year students. The primary rationale for including students in the clinical training period was that this group had completed their theoretical education and was actively involved in clinical practice, enabling them to evaluate the study variables in a meaningful, reliable, and sufficient manner. All medical students were invited to participate in the study (150 fourth-year, 130 fifth-year, and 130 sixth-year students). As participation was voluntary, the overall response rate was approximately 70.7%. Data were collected through face-to-face administration of a structured questionnaire that included items on age, gender, smoking status, and knowledge regarding smoking-related lung diseases. The data collection tool was a structured questionnaire developed by the researchers based on a review of the relevant literature and in accordance with the objectives of the study. To ensure content validity, the questionnaire was evaluated by experts in the field, and necessary revisions were made based on their feedback to finalize the instrument.

### Ethical Approval:

Ethical approval for the study was obtained from the Harran University Clinical Research Ethics Committee (Decision No: HRÜ/24.17.28; Date: November 4, 2024).

### Statistical analysis:

A statistical analysis of the recorded data was completed using IBM SPSS 25.0. Descriptive statistics for continuous variables were expressed as mean and standard deviation, while categorical variables were presented as numbers and percentages. Comparisons of categorical variables between groups were performed using Chi-square tests. p < 0.05 was accepted as a statistically significant difference between groups.

## RESULTS

The sociodemographic characteristics and smoking status of the students are shown in Table-I. Smoking prevalence was lowest among fourth-year students and highest among sixth-year students. Overall, 30.3% of the students were current smokers. When analyzed by gender, 75% of the smokers were male, whereas 25% were female.

**Table-I:** Sociodemographic characteristics and smoking status of the students.

Group	Age (mean ± SD)	Gender (Female/Male, %)	Marital status (Single/Married, %)	Current smokers (%)
4th year (n = 120)	23.14 ± 2.05	49.2 / 50.8	95.0 / 5.0	21.7
5th year (n = 80)	23.78 ± 1.22	43.8 / 56.2	97.5/ 2.5	32.5
6th year (n = 90)	24.23 ± 1.82	47.8 / 52.2	97.8 / 2.2	40.0
Total (n = 290)	23.57 ± 1.82	47.2 / 52.8	96.5 / 3.5	30.3

The distribution of students indicating an association between smoking and lung diseases across academic years is shown in Table-II. In our study, knowledge about the relationship between smoking and lung cancer was the highest, whereas awareness of its association with tuberculosis was the lowest.

**Table-II:** The relationship between students’ cigarette use and lung diseases.

Group	Lung cancer (%)	COPD (%)	Asthma (%)	Pneumonia (%)	Tuberculosis (%)	Interstitial Lung Disease (%)	Pneumothorax (%)
4th year (n = 120)	93.3	93.3	73.3	59.2	42.5	59.2	42.5
5th year (n = 80)	93.8	95.0	62.5	56.3	36.3	63.8	50.0
6th year (n = 90)	94.4	92.2	72.2	68.8	46.7	68.9	68.9
Total (n = 290)	93.8	93.4	70.0	61.4	42.1	63.4	52.8

When evaluated across different year groups, the knowledge level regarding the association of smoking with COPD and lung cancer was above 90% in all groups, with no significant differences between classes (p>0.05 ). Similarly, no significant differences were observed among the classes in recognizing the association of smoking with asthma, interstitial lung diseases, tuberculosis, or pneumonia (p>0.05). However, awareness of the relationship between smoking and pneumothorax was highest among sixth-year students, and a statistically significant difference was found between fourth and sixth year students (p = 0.003).

## DISCUSSION

Tobacco use is widespread in societies, and addiction to tobacco and tobacco products is prevalent worldwide. The use of tobacco products kills nearly 50% of its users. Globally, approximately 80% of tobacco users (1.1 billion) live in low-income countries. Ongoing global efforts have raised awareness about the negative effects of tobacco use, and population-level public health programs have supported smoking cessation for years.^6^

As in many countries worldwide, several studies have been conducted in our country regarding smoking prevalence and related factors among medical students.^7^-^9^ The main reasons for conducting these studies specifically on medical students include their critical role as future physicians in smoking cessation efforts and the demanding and lengthy medical education process, which may influence students’ tendency to smoke.^10^ In our study, we evaluated both the smoking status and the knowledge of smoking-related lung diseases among medical students.

In our study, the prevalence was slightly higher at 30.3%. In a multicenter study conducted across 12 medical schools in Turkey with the participation of 1217 students, the prevalence of smoking was reported as 28.5%.^7^ A study conducted in Karachi, Pakistan, showed that the smoking rate among male doctors and healthcare personnel working in tertiary hospitals was high, while this trend was low among female healthcare workers.^11^ In our study, the rate of smoking was considerably higher among male students compared to female students. Consistent with the findings of our study, a study conducted at Gazi University Faculty of Medicine reported significantly higher smoking rates among male students compared to female students.^8^ In our study, smoking prevalence increased as students progressed through their medical education. Similarly, a study conducted across 45 countries, including Türkiye, demonstrated that smoking prevalence among medical students increased with higher academic year levels.^9^

Chronic obstructive pulmonary disease (COPD) is characterized by irreversible airflow limitation. Although COPD is both preventable and treatable, it is believed to be associated with abnormal inflammatory responses of the lung to harmful gases and particles. Tobacco smoking is the leading risk factor, with nearly 80% of COPD patients being smokers. There is a dose-dependent relationship between smoking and COPD, meaning the risk is higher in heavier smokers. Both the duration and amount of smoking are important, but smoking duration is considered more significant in COPD risk. Passive smoke exposure also increases COPD risk.^2^ In our study, 93.4% of students associated smoking with COPD. Although this rate is high compared to rates found in other diseases, it is expected to be higher among clinical-year medical students. For example, in a study conducted by Mohigefer et al. among final-year medical students, only 1.5% of students did not consider tobacco use as the primary risk factor for COPD.^12^

Tobacco use is the most common preventable cause of cancer and is thought to account for about 30% of all cancers. Approximately 94% of lung cancers are attributed to tobacco, and smokers are estimated to have a 20-fold higher risk compared to non-smokers.^2^ In our study, lung cancer was the disease most frequently associated with smoking (93.8%). However, considering their medical education and future role as health educators, the level of knowledge should ideally be even higher. For example, in a study conducted by İnnabi and colleagues, 97.8% of students knew that smoking causes lung cancer.^13^

Tobacco has numerous negative effects on asthma. Early-life exposure to secondhand smoke predisposes to asthma, while in asthmatic patients, smoking exacerbates bronchial hyperresponsiveness and reduces response to corticosteroid therapy.^14^ In our study, 70% of students recognized smoking as a risk factor for asthma—a considerably lower percentage compared to COPD and lung cancer.

Interstitial lung diseases (ILDs) represent a broad spectrum of conditions characterized by diffuse parenchymal lung involvement and impaired gas exchange. Smoking-related ILDs include respiratory bronchiolitis-associated ILD (RB-ILD), Pulmonary Langerhans Cell Histiocytosis (PLCH), and desquamative interstitial pneumonia (DIP). Tobacco exposure causes diverse pathological changes in lung parenchyma and bronchioles.^15^ In our study, 63.4% of students associated smoking with ILDs. This relatively low awareness may be due to the rarity of these diseases and the limited time allocated to ILDs in medical curricula.

Tobacco smoke induces inflammation, fibrosis, increased mucosal permeability, and epithelial injury in alveoli and bronchioles, as well as impaired mucociliary clearance and immune dysfunction. Smoking has been identified as a major risk factor for community-acquired pneumonia (CAP), with studies reporting that smoking accounts for approximately 32.4% of CAP cases.^2^ In our study, only 61.4% of students linked smoking to pneumonia, suggesting the need for enhanced education on this topic.

Pneumothorax is defined as the accumulation of free air between the visceral and parietal pleura, commonly due to rupture of bullae or blebs. It has an incidence of 18–28 per 100,000 in men and 1.2–6 per 100,000 in women.^16^ Smoking is the strongest risk factor for primary spontaneous pneumothorax, with about 90% of patients being active smokers. The risk is 22 times higher in male smokers and nine times higher in female smokers compared to nonsmokers, with a dose-response relationship observed.^17^ Despite its strong association, only 52.8% of students in our study recognized the link between smoking and pneumothorax. This highlights the need for more comprehensive training on this subject in medical education.

Although the exact mechanisms are not fully understood, tobacco use is thought to impair T cell-mediated immunity and increase susceptibility to tuberculosis (TB).^2^ A study in Spain demonstrated that parental smoking significantly increased the risk of TB in children under 15 years of age, with risk increasing 4.2-fold if one parent smoked and 7.2-fold if both parents smoked. A systematic review of 34 studies (1988–2006) concluded that tobacco use increases the risk of TB, worsens TB-related mortality, and contributes to more severe forms of the disease.^18^ In our study, less than half of the students (42.1%) recognized the link between smoking and TB. Given TB’s public health importance, this lack of awareness underscores the need to emphasize smoking as a risk factor in TB-related education.

Our study revealed a relatively high prevalence of smoking among medical students, with higher rates in males and in senior academic years. Lung cancer was the most commonly recognized smoking-related disease, while tuberculosis was the least recognized. As a result of our literature review, we found that there are a limited number of studies evaluating medical students’ knowledge about smoking-related lung diseases. Our study is distinctive in that it addresses this topic using a comprehensive and holistic approach. Another important strength of our study is the inclusion of only students in the clinical training period. As this group reflects both preclinical knowledge and clinical experience, it allows for a more reliable assessment of knowledge regarding smoking-related lung diseases and enhances the clinical relevance of the study findings.

### Limitations:

The most important limitation of our study is that, because it is a survey study, participants may have provided limited and/or subjective information. However, to mitigate this situation, participants’ identities were not recorded. Another important limitation is that the study is cross-sectional. Future multicenter and longitudinal studies using standardized and validated tools are needed to better evaluate changes in smoking and smoking-related lung diseases knowledge levels during medical education.

## CONCLUSIONS

As future physicians, medical students must receive comprehensive training in smoking cessation to protect both their own health and that of their patients. They should be reminded of their responsibility to act as role models in smoking prevention and cessation. Medical educators, regardless of specialty, should avoid smoking themselves and provide sufficient education on smoking-related diseases not only during pulmonology clerkships but across all courses and clinical rotations.
